# Impact of Stagnation on the Diversity of Cyanobacteria in Drinking Water Treatment Plant Sludge

**DOI:** 10.3390/toxins14110749

**Published:** 2022-10-31

**Authors:** Farhad Jalili, Hana Trigui, Juan Francisco Guerra Maldonado, Sarah Dorner, Arash Zamyadi, B. Jesse Shapiro, Yves Terrat, Nathalie Fortin, Sébastien Sauvé, Michèle Prévost

**Affiliations:** 1 Department of Civil, Geological and Mining Engineering, Polytechnique Montréal, Montréal, QC H3C 3A7, Canada; 2 Faculty of Engineering and Information Technology, The University of Melbourne, Parkville, VIC 3010, Australia; 3 Department of Biological Sciences, University of Montréal, Montréal, QC H2V 0B3, Canada; 4 Department of Microbiology and Immunology, McGill University, Montréal, QC H3A 2B4, Canada; 5 McGill Genome Center, McGill University, Montréal, QC H3A 0G1, Canada; 6 National Research Council Canada, Energy, Mining and Environment, Montréal, QC H4P 2R2, Canada; 7 Department of Chemistry, University of Montréal, Montréal, QC H3C 3J7, Canada

**Keywords:** taxonomic cell counts, shotgun metagenomic sequencing, storage, sludge, microcystins

## Abstract

Health-related concerns about cyanobacteria-laden sludge of drinking water treatment plants (DWTPs) have been raised in the past few years. Microscopic taxonomy, shotgun metagenomic sequencing, and microcystin (MC) measurement were applied to study the fate of cyanobacteria and cyanotoxins after controlled sludge storage (stagnation) in the dark in a full-scale drinking water treatment plant within 7 to 38 days. For four out of eight dates, cyanobacterial cell growth was observed by total taxonomic cell counts during sludge stagnation. The highest observed cell growth was 96% after 16 days of stagnation. Cell growth was dominated by potential MC producers such as *Microcystis*, *Aphanocapsa*, *Chroococcus*, and *Dolichospermum*. Shotgun metagenomic sequencing unveiled that stagnation stress shifts the cyanobacterial communities from the stress-sensitive Nostocales (e.g., *Dolichospermum*) order towards less compromised orders and potential MC producers such as Chroococcales (e.g., *Microcystis*) and Synechococcales (e.g., *Synechococcus*). The relative increase of cyanotoxin producers presents a health challenge when the supernatant of the stored sludge is recycled to the head of the DWTP or discharged into the source. These findings emphasize the importance of a strategy to manage cyanobacteria-laden sludge and suggest practical approaches should be adopted to control health/environmental impacts of cyanobacteria and cyanotoxins in sludge.

## 1. Introduction

Cyanobacteria and cyanotoxins are a challenge in water resources worldwide that may affect drinking water quality [1,2,3,4,5]. Conventional treatment (flocculation, coagulation, sedimentation, and filtration) is widely applied to manage cyanobacterial cells and cell-bound metabolites in drinking water treatment plants (DWTPs) [6,7,8,9]. Although these processes can remove 60–99% of cyanobacterial cells from the intake water, they can cause an accumulation of cyanobacterial cells and cyanotoxins in the sludge [6,10,11,12,13]. It is reported that even low influent cell counts below 1000 cells/mL may lead to cyanobacterial accumulation in the sludge by up to 100-fold. Additionally, cyanotoxin concentrations detected below the detection limit (DL) in the intake water may increase 12-fold in the sludge [14,15].

Several studies have highlighted that *Microcystis aeruginosa*, *Dolichospermum circinale* (formerly *Anabaena circinalis*), *Oscillatoria* sp., and *Raphidiopsis raciborskii* (formerly *Cylindrospermopsis raciborskii*) can remain viable in the stored sludge for 2–12 days. During sludge storage, cells can undergo lysis leading to cyanotoxin release [6,12,16,17,18,19,20]. An investigation showed that concentrations of microcystins (MCs) and cylindrospermopsin in the stored sludge containing *Microcystis aeruginosa* and *Raphidiopsis raciborskii* cells remained 1.2–4 times higher than the maximum expected concentration calculated based on the cell toxin quota (if all cells release cyanotoxins) after 7–16 days [21]. This revealed that cells not only were able to survive in the stored sludge but also could retain the ability to grow. However, the authors mentioned that the underestimation of cell quota as well as further cell settlement from the supernatant might affect the results. Thus, cell growth of cyanobacteria in the stored sludge remains questionable. Recent studies reported that *Microcystis aeruginosa* and *Raphidiopsis raciborskii* cells could stay viable and proliferate in the sludge for around 35 days [22,23]. Additionally, cyanotoxin release can increase by up to 2.5 times during sludge storage.

Accumulation of cyanobacteria in the sludge could lead to technical problems and health issues. Some studies suggest that cyanobacteria-laden sludge should be disposed of within 2–4 days to minimize risks associated with metabolite release [24,25]. However, the negative impact of the sludge supernatant containing cyanobacteria and cyanotoxins was not investigated. One study reported that the recycling of cyanobacteria-laden sludge supernatant to the head of the plant caused a 40% increase in the cyanobacterial cell counts in the intake water [26]. In our previous study, we demonstrated the dynamics of bacterial and cyanobacterial diversity in the stored sludge and its impact on the sludge supernatant in a full-scale plant [27]. In addition, we showed the selective accumulation of *Microcystis* and *Dolichospermum* in the sludge after flocculation, coagulation, and sedimentation processes [27]. These results highlighted concerns about MC accumulation in the sludge and its impact on water quality when the sludge supernatant is recycled to the head of the DWTP or discharged into the source.

However, previous studies fall short of demonstrating the potential for cyanobacteria to grow during storage in the dark. Additionally, most of the previous sludge investigations were performed using cultured cyanobacteria, not natural cyanobacterial blooms. Secondly, the dynamic nature of a sludge holding tank operation precludes the quantification of the impact of storage on the growth of cyanobacterial cells. Furthermore, the survival and growth of cyanobacteria in the sludge are best investigated using shotgun metagenomic sequencing to observe microbial/cyanobacterial community dynamics during storage.

The general objective of this study was to assess the fate of cyanobacteria during sludge storage in a full-scale DWTP. The specific objectives were to (i) validate cyanobacterial cell growth during sludge storage, (ii) study the dynamics of the cyanobacterial compositions in the stored sludge under controlled conditions, (iii) investigate the most resistant and susceptible cyanobacterial genera during sludge storage, and (iv) study the potential health impact (i.e., cyanotoxin release) of the genera surviving sludge storage.

To the best of our knowledge, this is the first descriptive study on the fate of natural cyanobacteria-laden sludge during stagnation (storage) in a full-scale DWTP using taxonomic cell counts, shotgun metagenomic sequencing, MC measurement, and physico-chemical parameter quantification.

## 2. Results and Discussions

### 2.1. Overview of Microbial/Cyanobacterial Diversity, Sludge Characteristics, and Microcystin Concentrations

**Figure 1:**
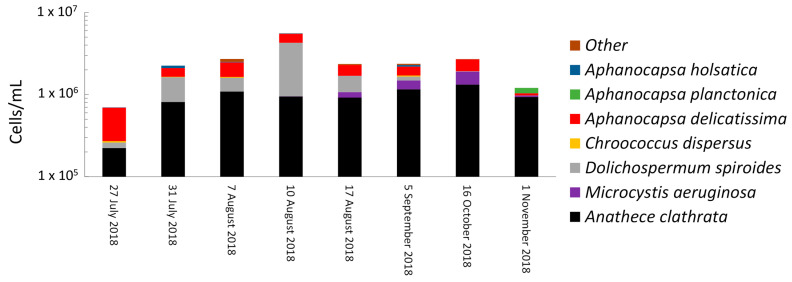
Cyanobacterial taxonomic cell counts in the sludge before stagnation. Other is less dominant species (<5%) including *Anathece smithii*, *Microcystis wesenbergii*, *Dolichospermum circinale*, *Dolichospermum planctonicum*, *Merismopedia tenuissima*, *Merismopedia minima*, *Merismopedia punctata*, *Pseudanabaena limnetica*, *Coelosphaerium kuetzingianum*, *Aphanizomenon issatschenkoi*, *Aphanizomenon flos-aquae*.

Taxonomic cell counts in the sludge varied from 0.7 × 10^6^ cells/mL (min. on 27 July 2018) to 5.6 × 10^6^ cells/mL (max. on 10 August 2018) (Table 1). *Anathece clathrata* (formerly *Aphanothece clathrata,* 17–77%), *Aphanocapsa delicatissima* (5–60%), *Dolichospermum spiroides* (formerly Anabaena spiroides, 0–59%) and *Microcystis aeruginosa* (0–21%) were predominant in the sludge samples during sampling dates (Figure 1). Overall, taxonomic cell counts in the sludge progressively increased from 27 July (0.7 × 10^6^ cells/mL) to 10 August (5.6 × 10^6^ cells/mL), then progressively decreased to 1.2 × 10^6^ cells/mL on 1 November at the end of the sampling campaign (Table 1). The highest total cell counts on 10 August corresponded with a high level of *Dolichospermum spiroides* (3.3 × 10^6^ cells/mL) and a low level of *Microcystis aeruginosa* (7.0 × 10^3^ cells/mL) (Figure 1). Interestingly, the highest cell counts of *Microcystis aeruginosa* (5.8 × 10^6^ cells/mL) detected on 16 October corresponded with a low level of *Dolichospermum spiroides* as 1.8 × 10^4^ cells/mL; this trend was already observed in this studied intake water [27,28].

**Figure 2:**
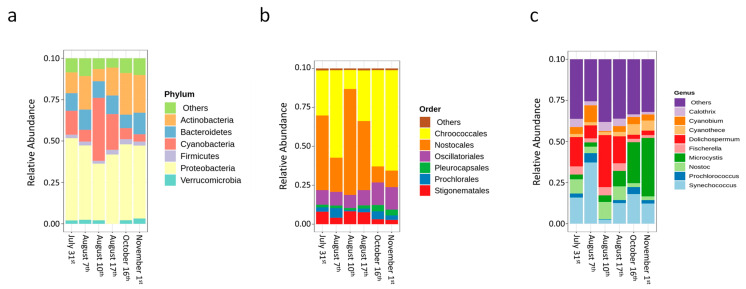
(**a**) Microbial communities at the phylum level, (**b**) Cyanobacterial communities at the order level, and (**c**) Cyanobacterial communities at the genus level in the sludge samples before stagnation.

Shotgun metagenomic sequencing revealed that Proteobacteria (35–52%), Cyanobacteria (5–39%), Actinobacteria (8–26%), and Bacteroidetes (8–14%) were the predominant phyla (Figure 2a) in the sludge and during the sampling campaign. Cyanobacteria reached its highest relative abundance level on 10 August (Figure 2a). This is in accordance with taxonomic cell counts (Figure 1). Nostocales (e.g., *Dolichospermum*) was the most abundant order on 31 July (47%), 10 August (76%), and 17 August (44%), whereas Chroococcales (e.g., *Microcystis*) was predominant on 7 August (57%), 16 October (61%), and 1 November (64%) (Figure 2b). Meanwhile, Oscillatoriales, Stigonematales, Prochlorales, and Pleurocapsales were detected in a low relative abundance (3–14%). At the genus level, *Synechococcus* (2–37%), *Microcystis* (3–36%), and *Dolichospermum* (2–32%) were predominant at all dates (Figure 2c). In addition, *Cyanobium* (<10%), *Nostoc* (<10%), *Calothrix* (<6%), *Cyanothece* (<4%), *Fischerella* (<4%), and *Prochlorococcus* (<4%) were detected in a lower relative abundance. Previously, at the same studied DWTP, we highlighted the selective accumulation of cyanobacteria at the genus and species levels by both shotgun metagenomic sequencing and taxonomic cell counts following conventional processes (flocculation/coagulation/sedimentation) [27]. However, the impact of stagnation was not systematically assessed to investigate the potential growth, lysis, and selective survival of cyanobacterial cells.

Although turbidity (171–920 NTU), total suspended solids (TSS, 716–3394 mg/L), total volatile solids (TVS, 367–1230 mg/L), and dissolved organic carbon (DOC, 3.4–9.8 mg/L) varied widely (Table 1), there was a significant association between pH and cyanobacterial community in the sludge (*p* < 0.05) (Figure S2). Values of pH varied from 6.74 (on 1 November) to 7.54 (on 7 August) (Table 1). In fact, on 7 August, the pH values of the incoming water, settled water, and sludge were in the high range of pH values for the studied period, with 7.05–7.94 in the intake water and 6.49–6.99 in the clarified water. On that day, *Synechococcus* was the most abundant genus (Figure 2c). The growth rate increase of *Synechococcus* sp. *WH7803* at pH values of 7–8 in artificial seawater was reported by Traving et al. [29].

Total MC concentrations remained below 239 ng/L from 27 July to 17 August (Table 1). Later in the season (5 September and 16 October), total MCs increased markedly to 1083 and 7413 ng/L, respectively; most were cell-bound (88% on 5 September and 96% on 16 October). This increase corresponds to elevated *Microcystis aeruginosa* cell counts on these two days (3.3 × 10^5^ cells/mL to 5.8 × 10^5^ cells/mL) (Figure 1). Total MCs decreased to 579 ng/L on 1 November in which 40% and 60% were cell-bound and dissolved, respectively (Table 1). Similarly, *Microcystis aeruginosa* cell counts remarkably deceased (3.8 × 10^4^ cells/mL) on this date (Figure 1).

### 2.2. Fate of Cyanobacterial Cells in the Sludge during Stagnation

**Figure 3:**
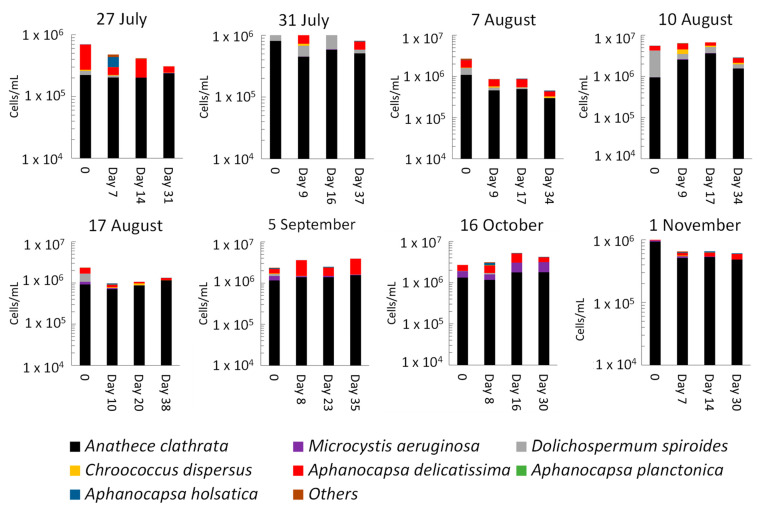
Taxonomic cell counts after sludge stagnation, 0: before stagnation. Other: see Figure 1.

Figure 3 and Figure S1 show the dynamics of taxonomic cell counts of sludge stored in the dark for up to 38 days. Overall, total cell counts increased for four out of eight sampling dates on 31 July, 10 August, 5 September, and 16 October, although the dynamics showed inconsistent yet interesting trends. Taxonomic cell counts during stagnation do not follow a consistent trend either by species or by duration of stagnation. Considering the fate of cyanobacterial species during stagnation, the results strongly suggest that several species grew during sludge stagnation on 27 and 31 July, 10 and 17 August, 5 September, 16 October, and 1 November. This was observed for several species including *Microcystis aeruginosa*, *Anathece clathrata*, *Aphanocapsa delicatissima* and *Aphanocapsa holsatica*, *Chroococcus disperses*, and *Dolichospermum spiroides.*

The highest cell count growth was observed on 16 October. For this sample, total cell counts clearly increased from 2.7 × 10^6^ cells/mL (before stagnation) to 3.1 × 10^6^ cells/mL (after 8 days of stagnation), 5.3 × 10^6^ cells/mL (after 16 days of stagnation), and 4.3 × 10^6^ cells/mL (after 30 days of stagnation). *Aphanocapsa holsatica* (4200%), *Dolichospermum spiroides* (582%), *Aphanocapsa delicatissima* (164%), *Microcystis aeruginosa* (134%), and *Anathece clathrata* (35%) had the highest contributions to this trend (Figure 3). Observations of total cell counts on 31 July, 10 August, 5 September, and 16 October provide clear evidence of growth during stagnation of the sludge in the dark for the first time. Concerns about cyanobacterial growth during stagnation have been recently raised by several authors but have not been demonstrated [21,22,23]. The fate of cyanobacterial cells in the sludge is complex and various environmental conditions (e.g., presence of nutrients) can contribute to their dynamics [22,23,27]. In our previous study on the same DWTP, we hypothesized that sludge storage time is an important parameter affecting cyanobacterial diversity and dynamics in the sludge [27]. Current findings point to the importance of sludge storage in terms of cyanobacterial growth potential.

**Figure 4:**
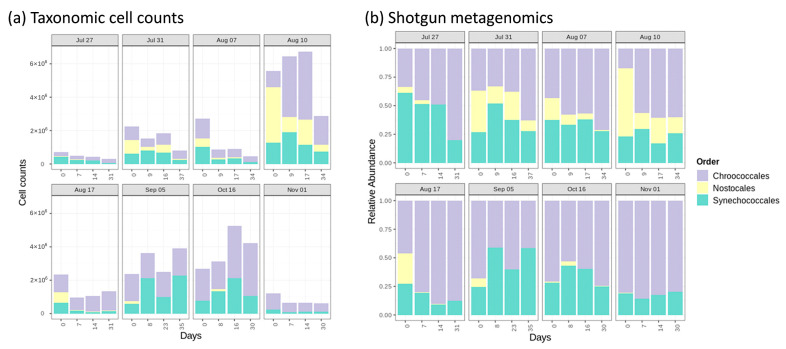
(**a**) Taxonomic cell counts, (**b**) Cyanobacterial community using shotgun metagenomic sequencing; before and after sludge stagnation at the order level, 0: before stagnation. Only the predominant orders Chroococcales, Synechococcales, and Nostocales are shown.

Considering cell counts at the order level, cell survival and growth were mostly observed within Synechococcales (e.g., *Aphanocapsa*) and Chroococcales (e.g., *Microcystis*) (Figure 4a). In contrast, Nostocales (e.g., *Dolichospermum*) most often markedly declined during stagnation, with some exceptions on 31 July (day 9 to 16), 10 August (day 9 to 17), and 16 October (before stagnation to day 9). Shotgun metagenomic sequencing showed that the relative abundance of Nostocales decreased in all stagnated samples as compared to the time zero point (before stagnation) (Figure 4b). The sole exception was 16 October, when the relative abundance of Nostocales increased slightly from the time zero point to day 8. The relative abundance of Chroococcales and Synechococcales remained either constant or increased on most of the dates except 27 July and 17 August (for Synechococcales) and 5 September (for Chroococcales). The only consistent trend is observed for Nostocales: when abundant, a clear decrease in the relative abundance was observed in almost all samples.

Overall, the variable trends are confirmed by both shotgun metagenomic sequencing and taxonomic cell counts at the order level, revealing the persistence of Synechococcales and Chroococcales as well as the sensitivity of Nostocales during stagnation (Figure 4b). Since the persistent genera are mostly MC producers [30,31], these findings emphasize the necessity of cyanobacteria-laden sludge management.

Non-concordance of taxonomic cell counts and shotgun metagenomic sequencing results are observed at the genus level (Figure 3 and Figure S3). For instance, *Aphanocapsa* including *delicatissima*, *holsatica,* and *planctonica* were detected by taxonomic cell counts, while they were not detected by shotgun metagenomic sequencing. Additionally, *Synechococcus* was detected by shotgun metagenomics, while it was not counted by taxonomic cell counts (Figure 3 and Figure S3). This non-concordance was reported in the previous investigations at the genus and species levels [27,32,33]. This can be due to either taxonomic cell count drawbacks, such as the misidentification of morphologically similar species [27,33,34,35], or shotgun metagenomic challenges, including DNA extraction, sequencing, and library limitation [36,37,38,39]. The observed concordance in diversity trends and shifts at the order level caused by stagnation are especially noteworthy. By combining taxonomic cell counts and shotgun metagenomic sequencing results, it is possible to conclude on abundance and cell counts, not only on relative shifts in diversity.

**Figure 5:**
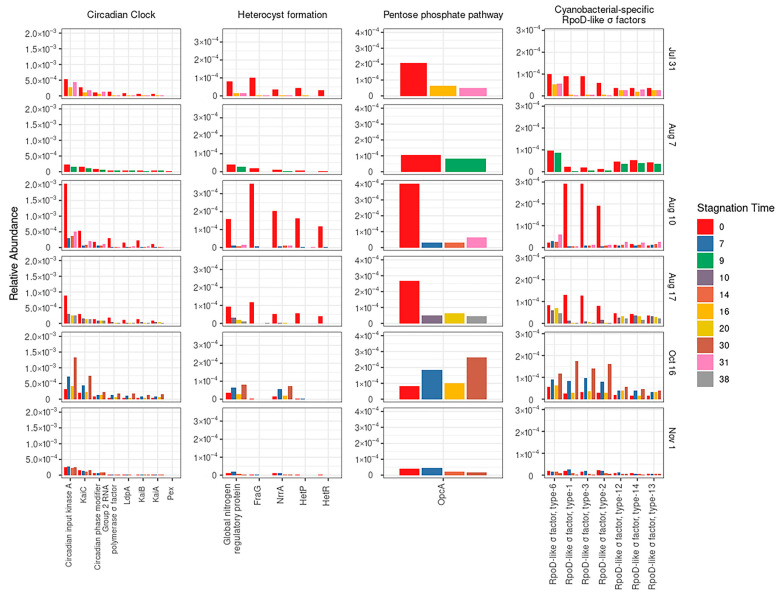
Relative abundance of selected cyanobacterial biomarker subsystems (level 4), before and after stagnation.

In order to track the functional cyanobacterial footsteps in response to stagnation, four cyanobacterial biomarkers (level 4 subsystems) were selected: “Cyanobacterial circadian clock”, “Heterocyst formation in Cyanobacteria”, “Transcription factors cyanobacterial rpoD-Like sigma factors”, and “Pentose phosphate pathway-opcA” [40,41,42]. The relative abundance of the selected biomarkers related to the “Cyanobacterial circadian clock” and “rpoD-like sigma factors” remarkably increased during stagnation on 16 October (Figure 5). Accordingly, they persisted on certain dates. This could be related to cell survival or growth during stagnation. “Heterocyst formation” is a cyanobacterial biomarker related to filamentous genera with heterocysts such as *Dolichospermum* and Nostoc (representatives of Nostocales) [42,43]. The relative abundance of this biomarker decreased during sludge stagnation. The exception was on the 16 October sample, where its relative abundance increased slightly, coinciding with the increase of *Dolichospermum* cell counts during stagnation (Figure 3). These findings are in line with our hypothesis about the vulnerability of Nostocales (e.g., *Dolichospermum*) and resistance of Chroococcales (e.g., *Microcystis*) as well as Synechococcales (e.g., *Aphanocapsa*) during sludge stagnation. In fact, *Microcystis* and *Aphanocapsa* have the ability to form a glycoprotein S-layer [44], protecting the cells against ecological stresses [45,46,47]. Interestingly, the “Pentose phosphate pathway (opcA)” marker gene, which is specific to dark heterotrophic growth [48], increases during stagnation, suggesting growth of cyanobacteria in the sludge holding tank (a dark place).

**Figure 6:**
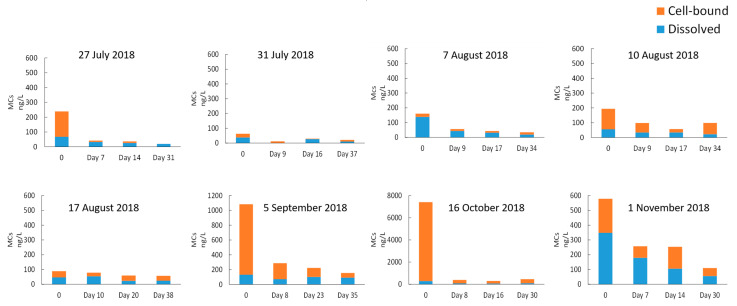
MC concentrations during sludge stagnation. 0: Before stagnation.

Although MC-producer genera such as *Microcystis*, *Aphanocapsa*, *Chroococcus*, and *Dolichospermum* grew during stagnation, cell-bound MCs generally decreased during the stagnation time (Figure 6).

The dynamics of cell-bound and dissolved MCs are influenced by: (i) the activation of the *mcy* genes in existing and newly grown cyanobacterial cells, and (ii) the rate of release and subsequent biodegradation of the released cell-bound MCs. In our previous study, we showed that sludge oxidation by potassium permanganate can play a role as oxidative stress and cause an increase in *mcyD* gene copy numbers in the oxidized sludge during storage [49]. Biodegradation of MCs has been shown for species representatives of Proteobacteria, Actinobacteria, and Firmicutes [50,51]. Moreover, some loss of MCs could be attributed to adsorption of dissolved MC onto the powdered activated carbon (PAC) injected into the intake water [27]. Since cell damage, cyanotoxin release, and cyanotoxin degradation occur simultaneously in the stored sludge, prediction of the cyanotoxin concentration in the sludge remains complex [21,27].

### 2.3. Cyanobacteria-Laden Sludge Management

While DWTP’s sludge is collected in a holding tank or lagoon to be disposed of, its supernatant can be discharged into the source water or a sewer, or be recycled to the head of the DWTP [26,52,53]. The presence of cyanobacteria and cyanotoxins in the recycled water can negatively affect the intake and treated water [26]. In this investigation, we showed that sludge storage can increase risks associated with the survival/proliferation of cyanotoxin producer species. This raises concerns in terms of the environmental and health risks associated with (i) supernatant handling (discharge or recycling), and (ii) residual (solids) disposal during periods of cyanobacterial blooms. Adjusting management strategies may include decreasing cyanobacterial loads in the sludge by optimizing the water treatment chain mainly through the addition of (i) pre-oxidation, (ii) supplementary treatment (e.g., separation and oxidation) to the recycling stream, (iii) sludge treatment (e.g., sludge oxidation as well as PAC injection), and (iv) restriction of land application of cyanobacteria-laden residuals.

## 3. Conclusions

1. Cyanobacterial growth, survival, and decay were quantified during sludge stagnation in the dark under controlled conditions. Longitudinal monitoring in summer and fall 2018 was conducted on sludge from a DWTP sludge holding tank. For four out of eight sampling dates, cyanobacterial cell growth was observed by total taxonomic cell counts during extended stagnation in the dark ranging from 7 to 38 days. The highest observed cell growth was 96% after 16 days of stagnation. The growth of cells was dominated by potential MC producers such as *Microcystis*, *Aphanocapsa*, *Chroococcus*, and *Dolichospermum*. Overall, up to 4200%, 1500%, 582%, and 134% cell growth was observed in potential MC-producer genera such as *Aphanocapsa*, *Chroococcus*, *Dolichospermum*, and *Microcystis*, respectively, during stagnation. Additionally, up to 35% cell growth was observed for non-toxic *Anathece*. Shotgun metagenomic sequencing revealed that sludge stagnation affected cyanobacterial diversity. Chroococcales (e.g., *Microcystis*) and Synechococcales (e.g., *Synechococcus*) were the most persistent orders, whereas Nostocales (e.g., *Dolichospermum*) was less resistant.
2. Sludge characteristics including cyanobacterial cell counts and MCs dynamically changed in the sludge. Amongst studied physico-chemical parameters, only pH showed a significant correlation (*p* < 0.05) with the cyanobacterial community in sludge.
3. Cyanobacterial biomarkers (level 4 subsystems) related to the “Circadian clock”, “rpoD-like sigma”, and “Pentose phosphate pathway” increased during stagnation, confirming cyanobacterial growth even in the dark. In contrast, the relative abundance of the “Heterocyst formation” biomarker related to filamentous genera declined in most of the stagnated samples.
4. Taxonomic cell counts, shotgun metagenomic sequencing, and cyanotoxin quantification provided consistent and complementary evidence regarding the quantification and dynamics of cyanobacteria in the stored sludge. This comprehensive investigation provides a sound basis to draft the best cyanobacteria-laden sludge management practices. Under the conditions tested, the persistence and/or growth of potential MC producers during storage raise the need to monitor cell counts and cyanotoxins in the sludge and its supernatant.
5. Handling cyanobacteria-laden sludge is a challenge as the presence of cyanobacterial toxins raises limitations for their safe disposal. Furthermore, cyanotoxin-laden sludge represents a risk to the intake and potentially treated water quality if the sludge supernatant is recycled to the head of the DWTP or is discharged into the source.

## 4. Materials and Methods

### 4.1. Description of the Studied Plant, Treatment Processes, and Sampling

A DWTP located in the southeast of Montreal was monitored from July to November 2018. The influent (intake water) of the DWTP is taken from Missisquoi Bay (Lake Champlain). The treatment chain includes powdered activated carbon (PAC) injection followed by coagulation, flocculation, sedimentation, filtration, and post-chlorination. The characteristics and operational data of the studied DWTP are presented in Table 2. The clarifier sludge is stored in a sludge holding tank (volume: 200 m^3^). The solid phase of the holding tank is transferred to a wastewater treatment plant (WWTP) for treatment. The supernatant of the holding tank is discharged into Missisquoi Bay (source). Sludge storage time in the holding tank varies from 7 to 38 days (prior to transferring to the WWTP). More details are explained in Jalili et al. [27]. Samples were taken from the bottom of the sludge holding tank (solid phase) on 27 and 31 July, 7 and 10 August, 5 September, 16 October, and 1 November 2018. 

### 4.2. Sludge Stagnation

Sludge stagnation was performed by storing the sludge samples in capped autoclaved polypropylene bottles in the dark and at room temperature (20 ± 2 °C) for 7 to 38 days. Stagnation times were selected based on the applied sludge storage times in the studied DWTP.

### 4.3. Sample Preparation

Sub-samples were prepared for taxonomic cell counts, shotgun metagenomic sequencing, cell-bound (intracellular) microcystins (MCs), dissolved (extracellular) MCs, dissolved organic carbon (DOC), and solids analysis.

The sub-samples of the time zero points (before stagnation) were prepared on-site at the DWTP using a portable laboratory prepared by our group at Polytechnique Montreal. An autoclaved 1-L propylene bottle was used to collect sludge samples and bring them back to Polytechnique Montreal for stagnation (storage). The bottles containing sludge samples were stored in a dark place during transportation.

A 40-mL vial was used for taxonomic cell counts. Lugol’s iodine was added to the taxonomic cell count sub-samples for preservation [27]. A 10-mL sample of sludge was collected in a sterile Falcon tube for shotgun metagenomic sequencing analysis. For cell-bound and dissolved MCs, samples were filtered using pre-weighted 0.45-μm GHP (hydrophilic polypropylene) membranes (Pall, Mississauga, ON, Canada). The filters were kept in the petri dish as cell-bound MCs and the filtrate was kept in 125-mL polyethylene terephthalate glycol (PETG) amber bottles (Thermo Fisher, Mississauga, ON, Canada) as dissolved MCs. DOC subsamples were prepared by filtration using pre-rinsed 0.45-μm membranes (PALL, Port Washington, NY, USA). The filtrate was collected and stored in 40-mL vials. All details are explained in our previous work [27].

### 4.4. Sample Analysis

#### 4.4.1. Taxonomic Cell Counts

Taxonomic cell counts were performed using an inverted microscope and a Sedgwick-Rafter chamber at magnifications of 10× and 40×. All details are explained in [27,49,54,55,56]. Taxonomic cell counts are widely applied for evaluation of cyanobacteria in water and sludge samples [7,11,14,15,21,22,27]. Measurement of chlorophyll-a (chla) and phycocyanin (PC) can provide more information about cell viability in the sludge. However, measurement of these parameters in the sludge is subjected to interferences due to elevated solids/turbidity using common approaches such as fluorometry techniques [57]. Therefore, taxonomic cell counts were selected for cell enumeration. Variability and reproducibility of taxonomic cell counts in our laboratory setting were investigated and the relative standard deviation was shown to be 4% [7]. Taxonomic cell counts were conducted by an experienced scientist considering cells in unicellular, aggregated, and filamentous forms.

#### 4.4.2. Dissolved Organic Carbon (DOC)

DOC analysis was performed according to the USEPA 415.1 method using a total organic carbon analyzer (Sievers Analytical Instruments, Boulder, CO, USA) [58].

#### 4.4.3. Microcystins (MCs)

MC analysis was performed using on-line solid-phase extraction ultra-high-performance liquid chromatography coupled to tandem mass spectrometry (on-line SPE-UHPLC-MS/MS). In brief, sample oxidation and quenching were performed using potassium permanganate, sodium periodate, and sodium bisulfite (Sigma Aldrich, Oakville, ON, Canada). Then, standard solutions of 4-phenylbutyric acid (50 ng/L) (Sigma Aldrich, Oakville, ON, Canada) and erythro-2-Methyl-3-methoxy-4-phenylbutyric acid (10 ng/L) (Wako Pure Chemicals Industries, Ltd., Osaka, Japan) were added to the samples. A 10-mL sample of the solution filtered on a 0.22-μm nylon filter (Sterlitech Corporation, Kent, WA, USA) was aliquoted for analysis using the Thermo EQUAN™ interface (Thermo Fisher Scientific, Waltham, MA, USA). An HTC Thermopal autosampler (CTC analytics, Zwingen, Switzerland) was applied to control the “in-loop” injection. A Thermo Hypersil Gold C18 column was applied for toxin separation. A Thermo TSQ QUANTIVA triple quadrupole mass spectrometer (Thermo Fisher Scientific) followed by UHPLC was applied for MS/MS detection. Water, methanol, and acetonitrile for HPLC were provided by Fisher Scientific (Whitby, ON, Canada). Formic acid (>95%), potassium carbonate, ammonium hydroxide (28–30% NH_3_), and ammonium acetate (≥99.0%) were purchased from Sigma Aldrich (Oakville, ON, Canada). All details are explained by Munoz et al. [59] and Roy-Lachapelle et al. [60].

#### 4.4.4. DNA Extraction, Shotgun Metagenomic Sequencing, Bioinformatics, and Statistical Analysis

Microbial and cyanobacterial dynamics were analyzed by shotgun metagenomic sequencing at the phylum, order, and genus levels. The number of reads of taxonomic data was normalized using the relative abundance.

Total nucleic acid was extracted by RNeasy PowerSoil Total RNA Kit (Qiagen, Toronto, ON, Canada) and DNA was eluted by the PowerSoil DNA Elution Kit (Qiagen, Toronto, ON, Canada). The Qubit V2.0 fluorometer (Life Technologies, Burlington, ON, Canada) was applied for the quantification of DNA. Sequencing was performed on the metagenomic libraries (Roche 454 FLX instrumentation with Titanium chemistry) by McGill University and Genome Quebec Innovation Centre. DNA sequencing was performed on the Illumina NovaSeq 6000 platform (S4 flow cells). The SolexaQA v3.1.7.1 program (default settings) was applied for the quality trimming of the raw reads [61]. FragGeneScan-Plus v3.0 and Cd-hit v4.8.1 were applied for the prediction of gene and protein fragments (90% similarity), respectively [62]. A similarity search was performed on a representative of each cluster using the M5nr database (https://github.com/MG-RAST/myM5NR, accessed on 17 September 2022) and the DIAMOND engine [63]. The best hits from the SEED Subsystems, KEGG, and COG databases [64,65,66] were used to determine the function fragments. Cyanobacterial functions were studied at Level 4 and the results were presented for the following biomarkers: “Cyanobacterial circadian clock”, “Heterocyst formation in Cyanobacteria”, “Transcription factors cyanobacterial rpoD-Like sigma factors”, and “Pentose phosphate pathway”. More details are presented in Moradinejad et al. [67].

Statistical analysis was done using R (3.6.2) and phyloseq (1.28.0) [68]. EasyCODA (0.31.1) was applied for the normalization of the taxonomic data [69]. The vegan package (2.5–6) (https://CRAN.R-project.org/package=vegan, accessed on 17 September 2022) was applied for beta diversity analysis based on the Euclidean distance. For the evaluation of the constrained variables, the redundancy analysis (RDA) was performed. Prior to the implementation of the model, the variance homogeneity was validated [70]. The permutation test was performed to select the significant (>95%) variables. All details can be found in our previous work [27].

#### 4.4.5. Solids Analysis

Total suspended solids (TSS) and total volatile solids (TVS) were measured according to the standard method 2540- Solids [71].

## Figures and Tables

**Table 1:** Sludge characteristics throughout sampling campaign. -: shotgun metagenomic sample not taken, *: shotgun metagenomic sample taken.

Sampling Date	Shotgun Metagenomic Sequencing	Taxonomic Cell Counts		MCs (ng/L)		DOC (mg/L)	pH	Turbidity (NTU)	TSS (mg/L)	TVS (mg/L)	Sludge Storage Time (d)
		Cells/mL ×10^6^	mm^3^/L	Cell-Bound	Dissolved						
27 July 2018	-	0.70	7.11	170.8	67.9	4.10	-	-	-	-	3
31 July 2018	*	2.25	147.4	24.9	37.9	3.60	7.05	201	716	367	7
7 August 2018	*	2.71	96.20	22.0	138.5	3.19	7.54	171	728	456	5
10 August 2018	*	5.57	608.9	138.2	55.8	5.20	6.8	258	1022	409	8
17 August 2018	*	2.35	138.3	41.6	46.6	3.35	7.12	327	1092	434	3
5 September 2018	-	2.37	52.76	951.8	131.2	9.80	6.81	701	1957	1230	6
16 October 2018	*	2.70	25.95	7129.0	284.2	3.46	6.87	2300	3394	1084	8
1 November 2018	*	1.21	4.42	230.8	348.2	2.76	6.74	225	740	546	6

**Table 2:** Water characteristics of the studied DWTP during the sampling campaign from July to October 2018.

Treatment Step	Parameters	July	August	September	October	Specifications
Raw water (RW)	Turbidity (NTU)	2.1–153.7	9.4–153.1	11.6–152.7	17.5–152.9	-
	pH	6.1–8.1	5.8–8.3	6.2–9.0	5.9–8.8	-
Clarifier (CW)	Turbidity (NTU)	0.01–20.1	0.30–20.0	0.33–10.2	0.01–10.3	PAC (wood-based): 1.5–27.0 mg/L, Coagulant: PAXL, 49–410 mg/L, Polymer: Hydrex (silicate), 0.05–0.1 mg/L Effective clarifier depth: 4.90 m, Max. sludge bed: 2.95 m, Hydraulic retention time: 1 h, Solid retention time: 48 h
	pH	6.1–7.2	6.7–7.2	6.2–7.1	6.6–7.2	
Dual sand-antrachite filter (FW)	Turbidity (NTU)	0.14–0.4	0.16–0.4	0.11–0.6	0.17–0.6	Retention time: 2 h
Treated water (TW)	Turbidity (NTU)	0.21–0.60	0.25–0.49	0.21–0.43	0.23–0.48	Injected chlorine: NaOCl, 1.3–6.0 mg/L
	pH	6.6–8.1	6.9–8.0	7.1–8.6	7.1–8.2	

## Data Availability

The sequencing data in this study are available in http://www.ncbi.nlm.nih.gov/bioproject/895678 (accessed on 17 September 2022).

## Supplementary Materials

The following supporting information can be downloaded at: https://www.mdpi.com/article/10.3390/toxins14110749/s1. Figure S1: Taxonomic cell count speciation (other than *Anathece clathrata*) after sludge stagnation, 0: before stagnation; Figure S2: (a) Impact of physico-chemical parameters on cyanobacterial communities at the order level. PC1: 70.3%, PC2: 28.3%. Only the significant parameter (pH) was shown (*p* < 0.05), (b) Cyanobacterial species grouped at the order level; Figure S3: Cyanobacterial community at the genus level during stagnation. d: stagnation day.
