# Supplementary material for: Impact of Stagnation on the Diversity of Cyanobacteria in Drinking Water Treatment Plant Sludge
Source: Toxins (Basel). 2022 Oct 31;14(11):749. doi: 10.3390/toxins14110749 (PMC9697381; doi:10.3390/toxins14110749)
Supplement: Supplementary file 1 [file toxins-14-00749-s001.zip › toxins-1950040-supplementary.pdf]

toxins

MDPI

Supplementary Materials: Impact of Stagnation on the Diversity of Cyanobacteria in Drinking Water Treatment Plant Sludge

Farhad Jalili, Hana Trigui, Juan Francisco Guerra Maldonado, Sarah Dorner, Arash Zamyadi, B. Jesse Shapiro, Yves Terrat, Nathalie Fortin, Sébastien Sauvé and Michèle Prévost

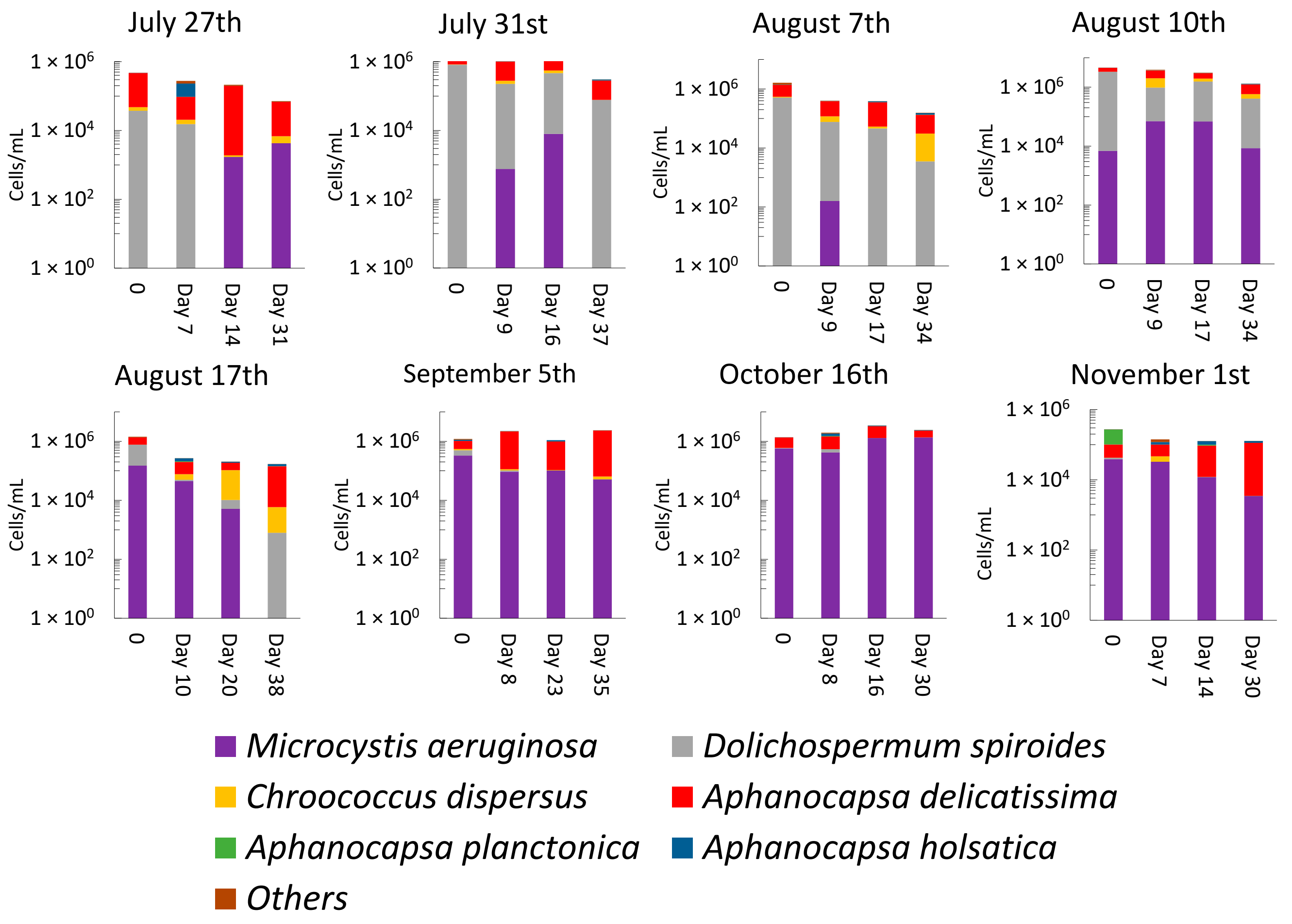

Figure S1. Taxonomic cell count speciation (other than *Anathece clathrata*) after sludge stagnation, 0: before stagnation. Other: see Figure 1.

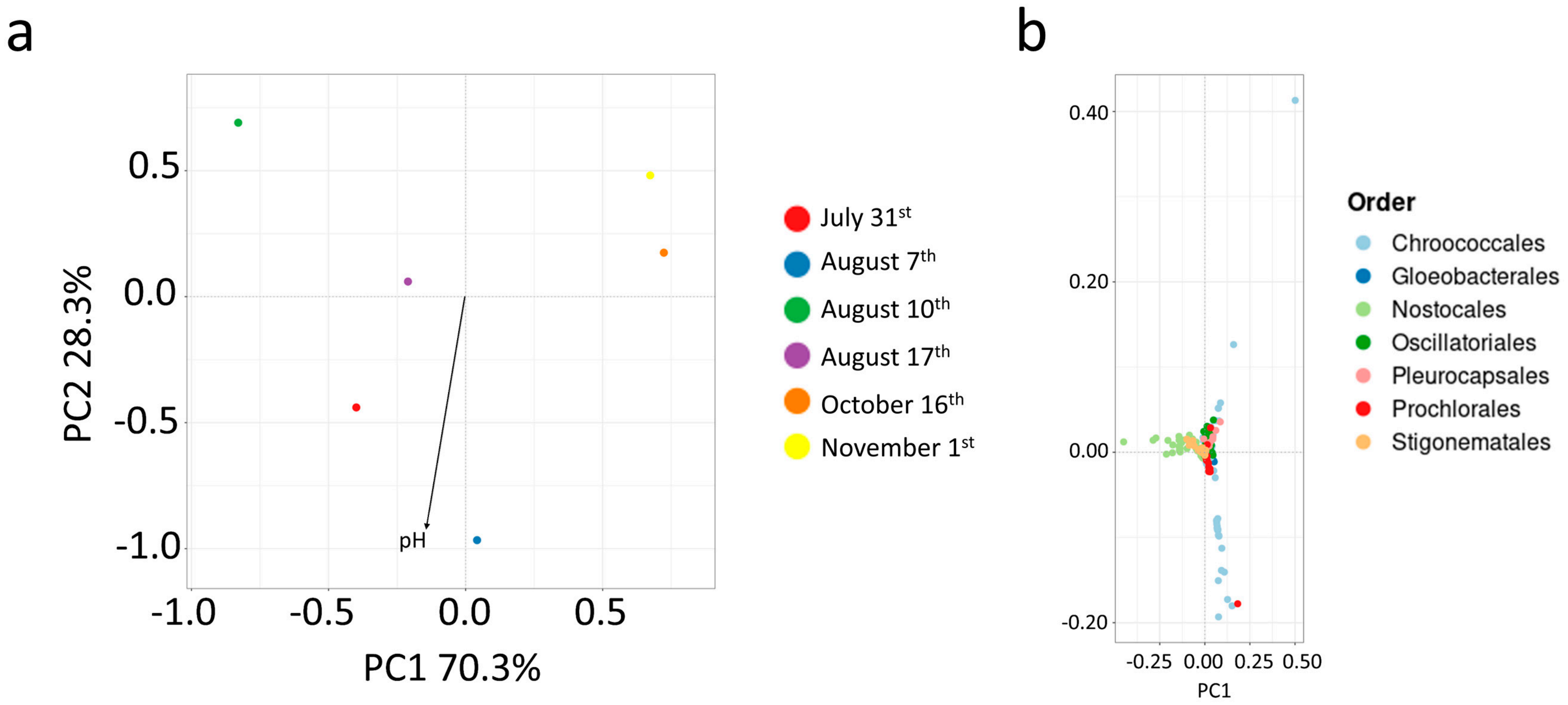

Figure S2. (a) Impact of physico-chemical parameters on cyanobacterial communities at the order level. PC1: 70.3%, PC2: 28.3%. Only the significant parameter (pH) was shown (p <0.05), (b) Cyanobacterial species grouped at the order level.

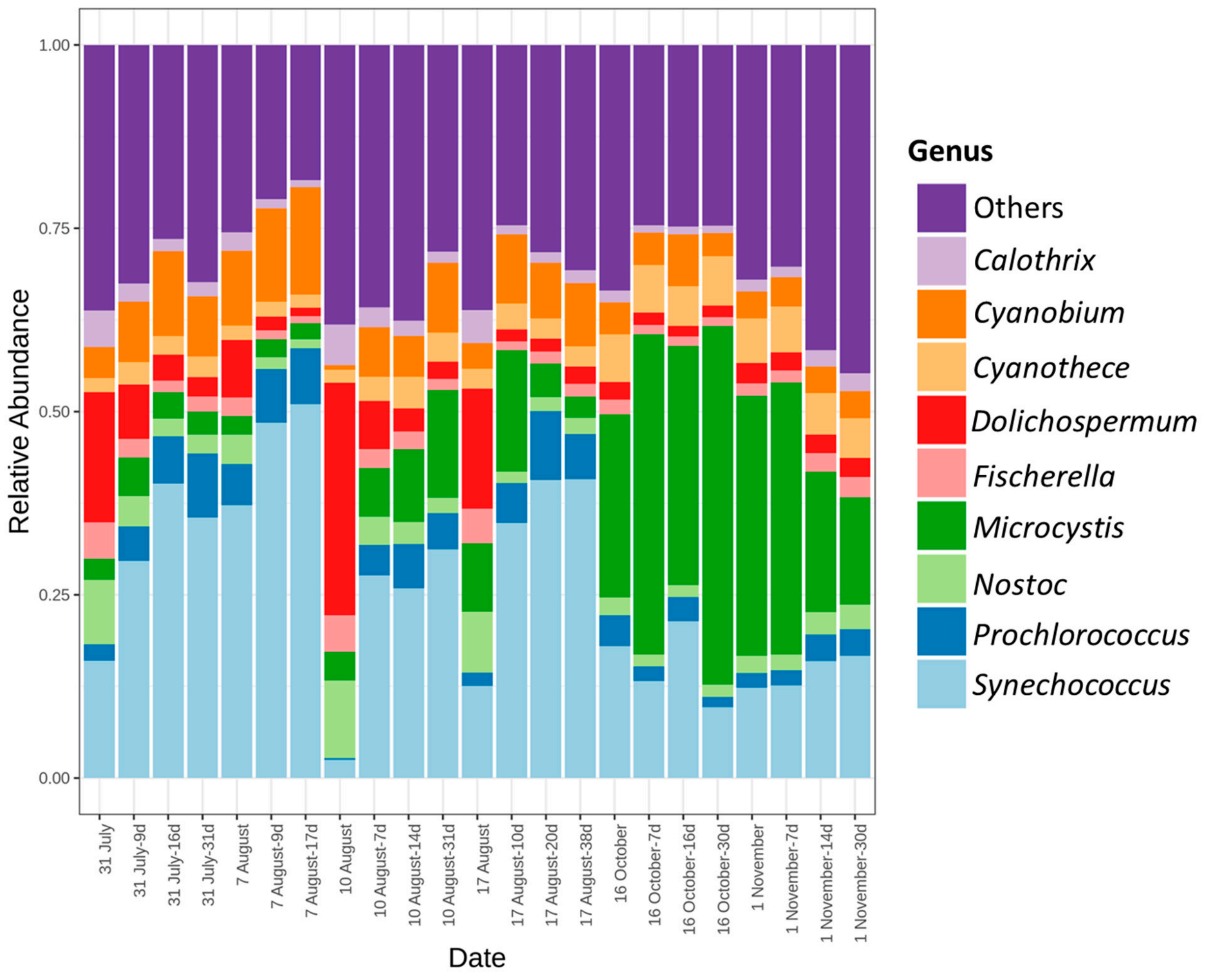

Figure S3. Cyanobacterial community at the genus level during stagnation. d:stagnation day.
